# Heat Stress Decreases Levels of Nutrient-Uptake and -Assimilation Proteins in Tomato Roots

**DOI:** 10.3390/plants6010006

**Published:** 2017-01-19

**Authors:** Anju Giri, Scott Heckathorn, Sasmita Mishra, Charles Krause

**Affiliations:** 1 Department of Environmental Sciences, University of Toledo, Toledo, OH 43606, USA; anjugiri1@gmail.com (A.G.); samishra@kean.edu (S.M.); 2 U.S. Department of Agriculture, University of Toledo, Toledo, OH 43606, USA; charles.krause@ars.usda.gov

**Keywords:** heat stress, high temperature, nutrients, nutrient-uptake proteins, *Solanum lycopersicum*, tomato

## Abstract

Global warming will increase root heat stress, which is already common under certain conditions. Effects of heat stress on root nutrient uptake have rarely been examined in intact plants, but the limited results indicate that heat stress will decrease it; no studies have examined heat-stress effects on the concentration of nutrient-uptake proteins. We grew *Solanum lycopersicum* (tomato) at 25 °C/20 °C (day/night) and then transferred some plants for six days to 35 °C /30 °C (moderate heat) or 42 °C/37 °C (severe heat) (maximum root temperature = 32 °C or 39 °C, respectively); plants were then moved back to control conditions for seven days to monitor recovery. In a second experiment, plants were grown for 15 days at 28 °C/23 °C, 32 °C/27 °C, 36 °C/31 °C, and 40 °C/35 °C (day/night). Concentrations of nutrient-uptake and -assimilation proteins in roots were determined using protein-specific antibodies and ELISA (enzyme-linked immunosorbent assay). In general, (1) roots were affected by heat more than shoots, as indicated by decreased root:shoot mass ratio, shoot vs. root %N and C, and the level of nutrient metabolism proteins vs. less sensitive photosynthesis and stomatal conductance; and (2) negative effects on roots were large and slow-to-recover only with severe heat stress (40 °C–42 °C). Thus, short-term heat stress, if severe, can decrease total protein concentration and levels of nutrient-uptake and -assimilation proteins in roots. Hence, increases in heat stress with global warming may decrease crop production, as well as nutritional quality, partly via effects on root nutrient relations.

## 1. Introduction

High-temperature stress, both from chronic or abrupt heating, is often a limiting factor for plant growth, development, and reproduction [1,2,3]. Both chronic and abrupt heat stress are expected to increase as a consequence of anthropogenically-driven global warming [4]. For example, in the mid-continental United States and Europe, the frequency, severity, and duration of heat waves is expected to increase in the future [5]. In many cases, it is expected that increases in extreme high-temperature events (e.g., heat waves) will affect plants more negatively than increases in average temperatures [6]. Heat stress decreases plant function in many ways, with negative effects on growth, photosynthesis, respiration, reproduction, water relations, and hormone production being especially well-studied [3,7,8,9,10].

Both shoots and roots are sensitive to heat-related damage, and roots are often as sensitive, or more, as shoots to a heat stress [7,11]. Further, roots are often subjected to high and potentially-stressful temperatures; e.g., when canopies are not closed and soil receives direct sunlight [12,13,14] or in cool-season species during hot summer months [15]. The tolerance of roots to heat stress scales with the mean temperatures of the habitats to which the species are adapted, such that optimal temperatures for root growth are lower in cool-season species, higher in warm-season species, and still higher in warm-desert plants [11]. For example, soil temperatures can exceed 30 °C in the top 5 cm of the soil profile in cool-season wheat fields in cool-temperate locations [15], 33 °C to 10 cm depth in oat fields in Texas [14], 35 °C–40 °C to 10 cm depth in a sub-tropical maize field [16], and 70 °C to 40 °C from the surface to 15 cm depth in deserts dominated by succulents [17]; in each of these cases, these soil temperatures exceed optimal temperatures for root growth for the respective species.

High-temperature stress reduces root growth, number, and mass [7], which affects the growth of aboveground tissue by restricting the supply of water and mineral nutrients, affecting production of hormones synthesized in roots and transported to shoots, and altering sink-source relationships between shoots and roots [7,8,9,18]. Relative to shoots, less research has examined the effects of heat stress on roots, and most of this past research had focused on root growth and carbon relations (especially respiration) [7]. Relatively little past research has investigated how heat stress affects plant nutrient relations [7,8,11,19], and most of this previous work has measured only heat effects on nutrient content or concentration. In addition, most of the past research on root heat stress has focused on chronic heat stress, but the responses of roots to chronic warming can differ from abrupt heat stress [11]; hence, the effects of abrupt heat stress on root nutrient-uptake is especially poorly understood.

Based on the few past studies, it is known that heat stress often decreases the concentration of nutrients in plant tissues or decreases the total content of nutrients in the plants, though effects can vary among nutrients and species [11]. Heat stress can also disrupt enzymes involved in nutrient metabolism (e.g., nitrate and ammonium assimilation) [20,21]. Decreases in nutrient acquisition with heat stress could potentially be caused by several factors, including a decrease in root mass or surface area and/or a decrease in nutrient uptake per unit root [19,22]. Decreases in nutrient uptake per unit root might be caused by depletion of labile C (total non-structural carbohydrate), and hence energy, in roots (e.g., due to a decrease in transport of shoot C to roots or an increase in root respiration) or by direct heat damage to roots [7], which might decrease the production or function of nutrient-uptake proteins. For most mineral nutrients, the bulk of their uptake is mediated by the activity of specific nutrient-uptake proteins, and uptake protein activity depends on both the concentration of uptake proteins per unit root, as well as the rate at which each protein works. However, almost nothing is known about the effect of heat stress on nutrient-uptake proteins in roots.

To investigate the effects of heat stress on nutrient uptake and nutrient-uptake proteins, we determined the effects of moderate to severe short-term heat stress in roots of tomato (*Solanum lycopersicum* L. cultivar Bigboy) on the concentration of the following key nutrient-uptake proteins: NRT1 and NRT2, the main low- and high-affinity nitrate transporters [23]; AMT1, the primary ammonium transporter [23]; PHT1, the primary root phosphorus transporter [24]; KT1, the main potassium transporter [25], FRO1, iron reductase, one of the two main Fe-uptake proteins in dicots [26]; and BOR1 and NIP5;1, the two main B transporters [27]. In addition, we also determined the effects of heat on the levels of key N assimilation enzymes: nitrate reductase (NR), glutamate dehydrogenase (GDH), glutamine synthetase (GS), and glutamate oxoglutarate amino transferase (GOGAT) [28]. Finally, we also measured the effects of heat on shoot and root growth, photosynthesis and stomatal conductance, and root total protein content, to determine how heat effects on nutrient uptake and nutrient-metabolism proteins relate to effects on root vs. shoot growth and function. Tomato was used as a model system because it is a warm-season moderately-thermotolerant species [29,30], originates from warm sub-tropical habitats that experience variable temperatures and abrupt heat stress [31], and has been used as a model in many heat stress studies [11]. In addition, given that the majority of tomato roots are in the top 10–20 cm of soil, soil temperatures can frequently exceed optimal levels in field-grown tomato (e.g., exceed 34 °C–35 °C at 10–15 cm, with optimal ca. 25 °C–27 °C) [32,33,34].

## 2. Results

### 2.1. Main Experiment

**Figure 1:**
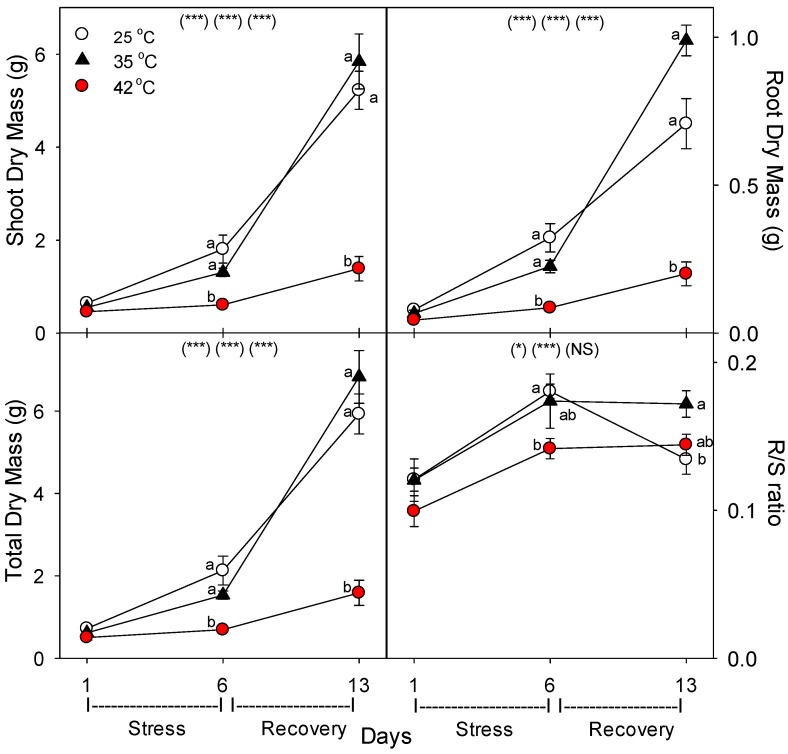
Effect of abrupt heat stress on biomass of tomato. Plants were grown at 25/20 °C day/night (control), and then subsets of plants were heat stressed at 35/30 or 42/37 °C day/night for one or six days (maximum root temperatures = 32 or 39 °C), and then returned to control conditions for seven days of recovery (=day 13). Values are means ± 1 SE for four independent replicates from each harvest (days 1, 6, 13). Within each variable, the significance of main treatment effects (temperature, day, temperature × day) is indicated in parentheses (* *p* < 0.05, ** *p* < 0.01, *** *p* < 0.001, NS = not significant). Different lowercase letters indicate significant difference among temperature treatments within each day.

Relative to control plants of the same age, both root and shoot biomass were decreased by the severe heat treatment, and root growth was more sensitive than shoot growth under severe heat stress, as indicated by the decline in root:shoot mass ratio at 42 °C (Figure 1). The biomass of moderately-stressed plants was only slightly (and non-significantly) decreased with six days of heating, and the biomass of these plants was slightly (and non-significantly) above unheated controls after the seven days recovery period under control conditions (i.e., by day 13). In contrast, relative to control plants of the same age, severely-stressed plants did not recover from heat within seven days, and their total biomass was 73% less than controls on day 13.

**Figure 2:**
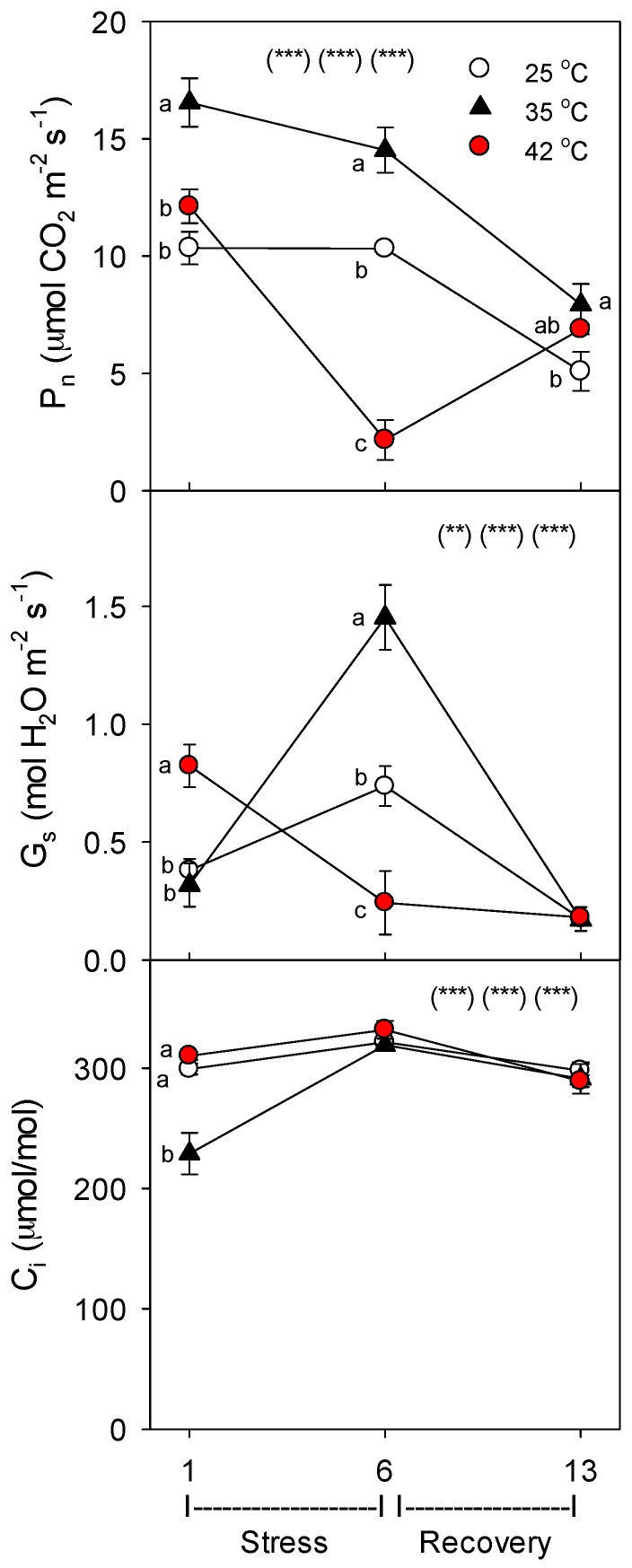
Effect of abrupt heat stress on net photosynthesis (P_n_), stomatal conductance (G_s_), and leaf internal CO_2_ concentration (C_i_) of tomato. Plants were grown at 25 °C/20 °C day/night (control), and then subsets of plants were heat stressed at 35/30 or 42 °C/37 °C day/night for one or six days (maximum root temperatures = 32 °C or 39 °C), and then returned to control conditions for seven days of recovery (=day 13). Values are means ± 1 SE for four independent replicates from each harvest (days 1, 6, 13). Statistics are as in Figure 1. * *p* < 0.05, ** *p* < 0.01, *** *p* < 0.001.

Net photosynthesis (P_n_) increased with moderate heat (35 °C), relative to control plants of the same age, but decreased with severe heat (day 6 only, with full recovery by day 13) (Figure 2). Severely-stressed plants opened their stomates more vs. controls (i.e., increased stomatal conductance, G_s_), on day 1 of heat stress, which is common [29] and can increase evaporative cooling, but had lower G_s_ on day 6 vs. controls; in moderately-stressed plants, G_s_ was increased on day 6, but similar to controls on day 1 (all treatments were similar on day 13). Except for day 1 (where 35 °C < controls or 42 °C), leaf internal carbon dioxide concentration (C_i_) was similar among the treatments, indicating that decreases in P_n_ were not caused by stomatal closure in heated plants, and thus were not caused by water stress (as similar to [30,35]).

**Figure 3:**
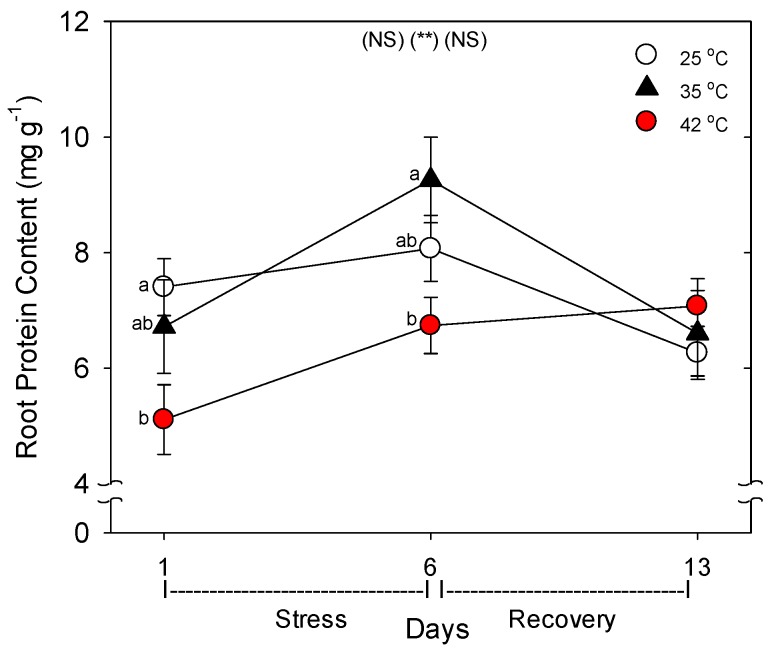
Effect of abrupt heat stress on protein content per gram (fresh weight) of roots. Plants were grown at 25 °C/20 °C day/night (control), and then subsets of plants were heat stressed at 35 °C/30 °C or 42 °C/37 °C day/night for one or six days (maximum root temperatures = 32 °C or 39 °C), and then returned to control conditions for seven days of recovery (=day 13). Values are means ± 1 SE for four independent replicates from each harvest (days 1, 6, 13). Statistics are as in Figure 1. ** *p* < 0.01.

**Figure 4:**
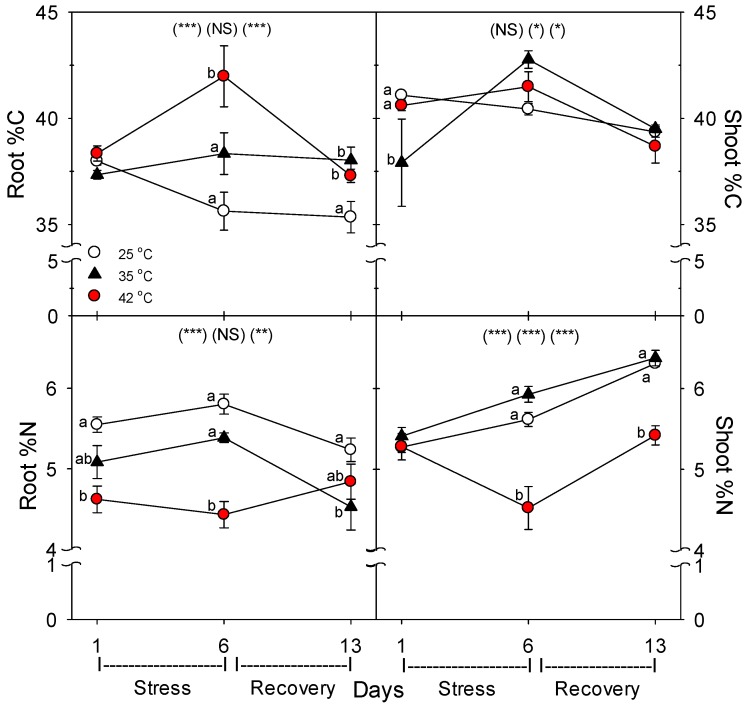
Effect of abrupt heat stress on the concentration of C and N in shoot (= leaves + stems) and roots of tomato (dry mass basis). Plants were grown at 25 °C/20 °C day/night (control), and then subsets of plants were heat stressed at 35 °C/30 °C or 42 °C/37 °C day/night for one or six days (maximum root temperatures = 32 °C or 39 °C), and then returned to control conditions for seven days of recovery (=day 13). Values are means ± 1 SE for four independent replicates from each harvest (days 1, 6, 13). Statistics are as in Figure 1. * *p* < 0.05, ** *p* < 0.01, *** *p* < 0.001.

**Figure 5:**
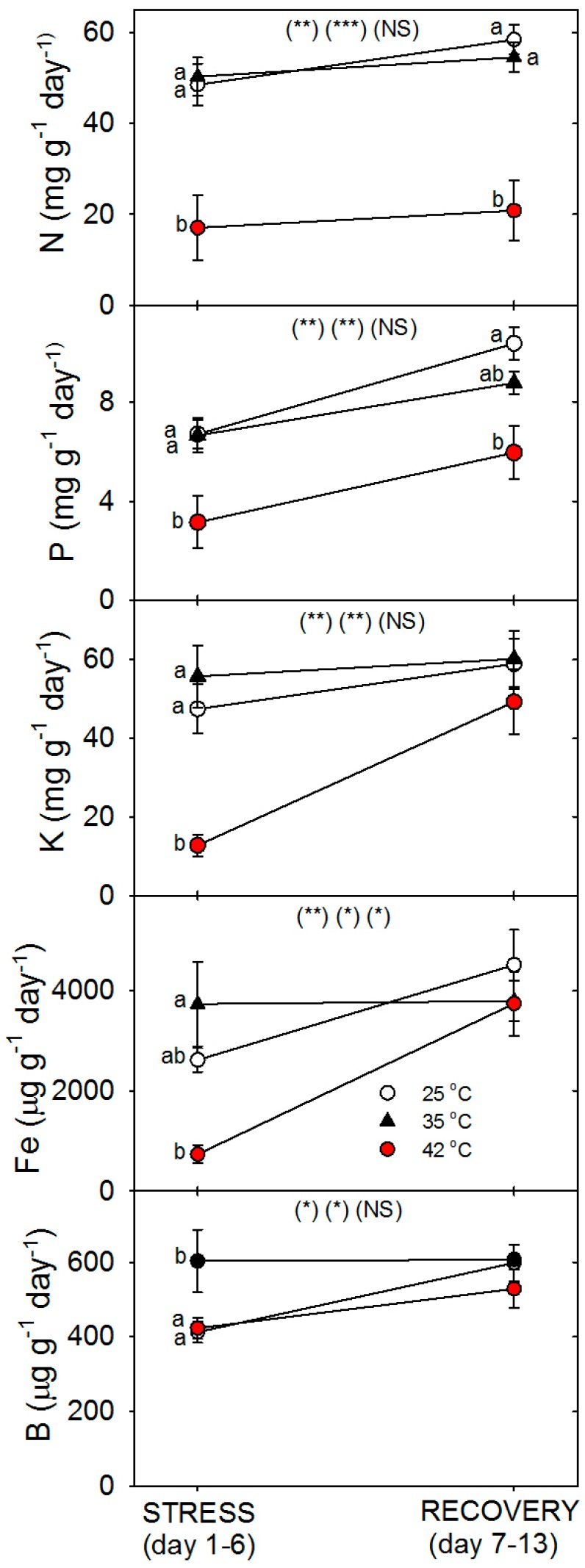
Effect of abrupt heat stress (days 1–6) and recovery (7–13) on the uptake rate of specific nutrients by roots of tomato (total mg for macronutrients and µg for micronutrients in the plant per g dry root per day). Plants were grown at 25 °C/20 °C day/night (control), and then subsets of plants were heat stressed at 35 °C/30 °C or 42 °C/37 °C day/night for one or six days (maximum root temperatures = 32 °C or 39 °C), and then returned to control conditions for seven days of recovery (=day 13). Values are means ± 1 SE for four independent replicates from each harvest (days 1, 6, 13). Statistics are as in Figure 1. * *p* < 0.05, ** *p* < 0.01, *** *p* < 0.001.

Relative to control plants of the same age, root protein content decreased only in plants heated to 42 °C, with full recovery by day 13 (Figure 3). Compared to control plants of the same age, the C concentration of roots was greater following six days of heating (especially at 42 °C), and this increase above control plants persisted after seven days of post-heat recovery, but there was no significant effect of heating on shoot %C, excluding on day 1, where heat decreased %C at 35 °C (Figure 4). In contrast to %C, %N in the roots tended to be less in heat-stressed plants relative to controls during the entire experiment (especially for 42 °C at days 1 and 6), while in shoots, heats effects on %N, compared to age controls, were significant only for 42 °C on days 6 and 13, wherein %N was lower. The uptake rate of macronutrients (NPK) and the micronutrient, Fe, by roots decreased with heat stress, compared to controls of the same age, but only at 42 °C (a non-significant decrease in K and Fe was observed at 35 °C) (Figure 5). Interestingly, B uptake rate did not decrease with heat, but rather increased at 35°C relative to other treatments (days 1–6). Notably, N and P uptake rate was still lower in severely-heat-stressed plants during recovery, whereas K and Fe uptake largely recovered by day 13, compared to age controls.

**Figure 6:**
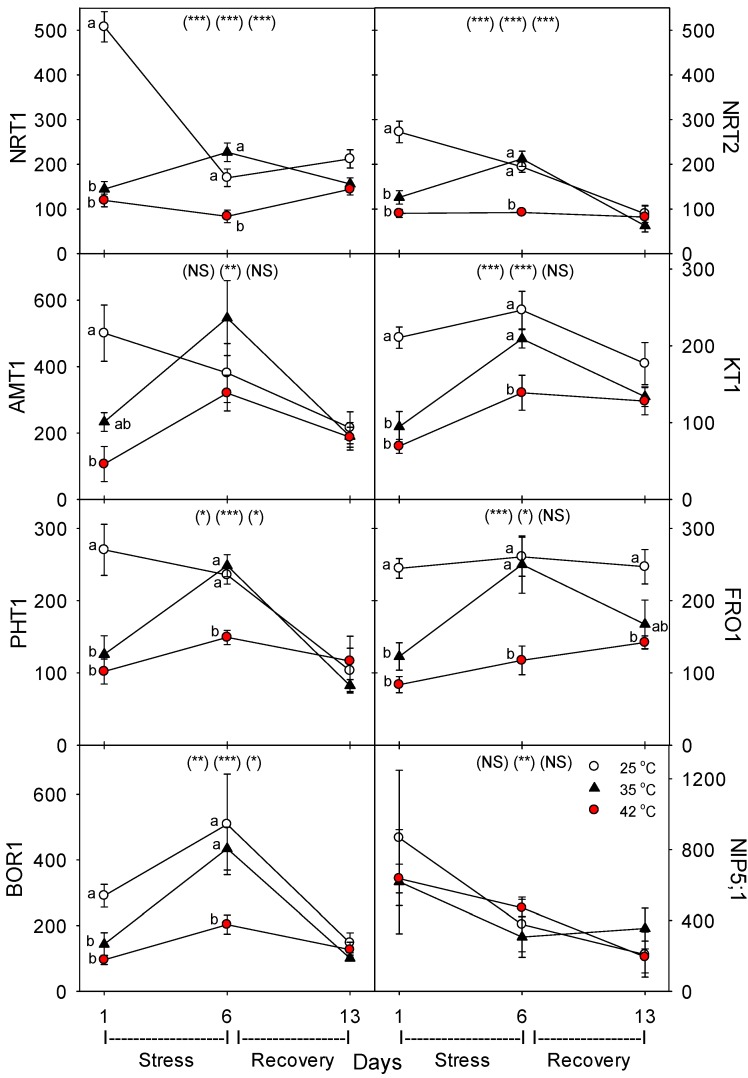
Effect of abrupt heat stress on relative levels of specific nutrient transport proteins (per g dry root) in roots of tomato: low-affinity NO_3_, NRT1; high-affinity NO_3_, NRT2; NH_4_, AMT1; K, KT1; P, PHT1; Fe, FRO1; B, BOR1 and NIP5;1. Plants were grown at 25 °C/20 °C day/night (control), and then subsets of plants were heat stressed at 35 °C/30 °C or 42 °C/37 °C day/night for one or six days (maximum root temperatures = 32 °C or 39 °C), and then returned to control conditions for seven days of recovery (=day 13). Values are means ± 1 SE for four independent replicates from each harvest (days 1, 6, 13). Statistics are as in Figure 1. * *p* < 0.05, ** *p* < 0.01, *** *p* < 0.001.

**Figure 7:**
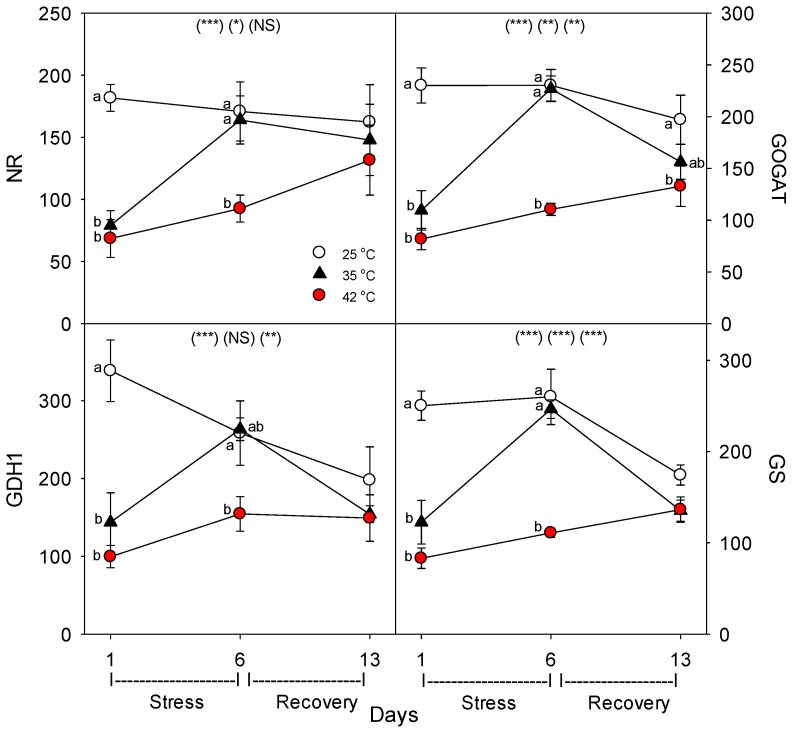
Effect of abrupt heat stress on relative levels of specific nutrient metabolism proteins (per g dry root) in roots of tomato: NR, nitrate reductase; GOGAT, glutamine oxoglutarate amino transferase; GDH1, glutamate dehydrogenase; GS, glutamine synthetase. Plants were grown at 25 °C/20 °C day/night (control), and then subsets of plants were heat stressed at 35 °C/30 °C or 42 °C/37 °C day/night for one or six days (maximum root temperatures = 32 °C or 39 °C), and then returned to control conditions for seven days of recovery (=day 13). Values are means ± 1 SE for four independent replicates from each harvest (days 1, 6, 13). Statistics are as in Figure 1. * *p* < 0.05, ** *p* < 0.01, *** *p* < 0.001.

**Figure 8:**
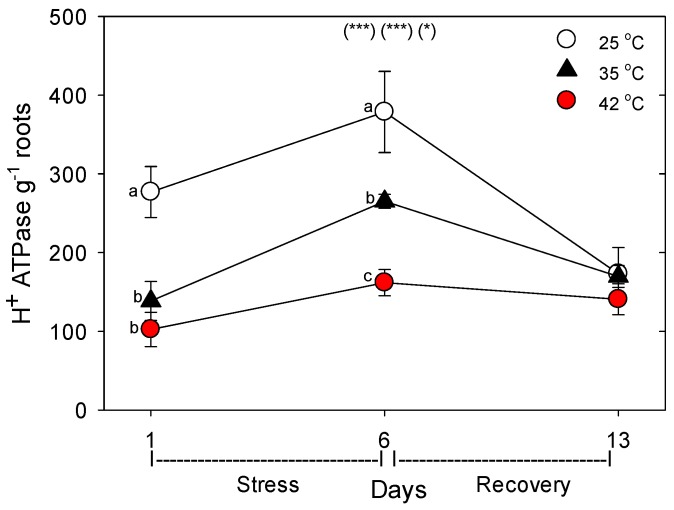
Effect of abrupt heat stress on relative levels of the plasmalemma H^+^-ATPases (per g dry root) in roots of tomato. Plants were grown at 25 °C/20 °C day/night (control), and then subsets of plants were heat stressed at 35 °C/30 °C or 42 °C/37 °C day/night for one or six days (maximum root temperatures = 32 °C or 39 °C), and then returned to control conditions for seven days of recovery (=day 13). Values are means ± 1 SE for four independent replicates from each harvest (days 1, 6, 13). Statistics are as in Figure 1. * *p* < 0.05, ** *p* < 0.01, *** *p* < 0.001.

Relative to control plants of the same age, heat stress (both 35 °C and 42 °C) initially decreased the concentration (per g dry root) of all the nutrient-uptake proteins we measured (NRT1, NRT2, AMT1, KT1, PHT1, FRO1, BOR1, and NIP5;1) (Figure 6, day 1). After six days of heat stress, the levels of nutrient uptake proteins in moderately-stressed plants were similar to unheated controls, but in severely-stressed plants, levels of these proteins remained below controls in all except for NIP5;1 and AMT1. After seven days of post-heat recovery, levels of these nutrient-uptake proteins had recovered to levels observed in control plants for all but FRO1 (with smaller non-significant decreases for NRT1 and KT1). Similar patterns, in heat-stressed plants compared to controls of the same age, were observed in the relative level of the nutrient assimilation proteins examined (NR, GOGAT, GDH, GS), with decreases in their levels following one day of heat stress, recovery of their levels in moderately-stressed, but not in severely-stressed, plants by day 6, and a tendency for near-complete recovery in both heat treatments by day 13 (Figure 7). As with the nutrient-uptake proteins, heat decreased levels of the plasmalemma H^+^-ATPase, compared to age controls, but decreased levels of this protein during the entire six days of heat treatment at both 35 °C and 42 °C; H^+^-ATPase levels recovered to control-plant levels by the end of the seven days post-heat recovery period (Figure 8).

### 2.2. Second Confirmatory Experiment

**Figure 9:**
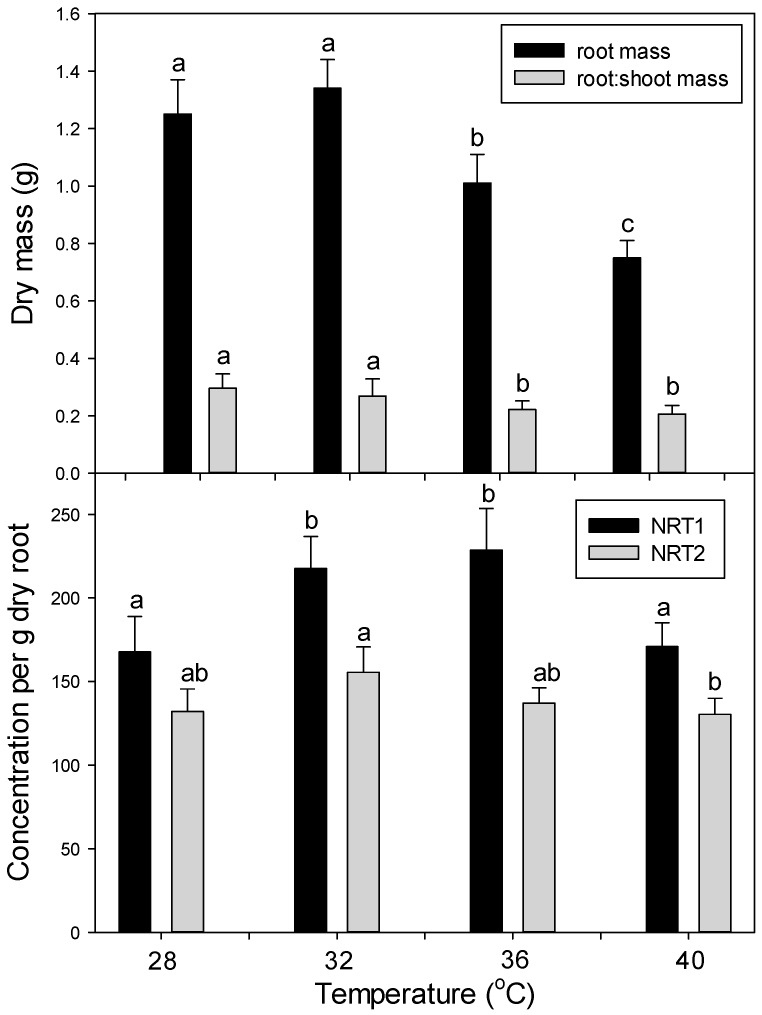
Effect of chronic heat stress on (top panel) plant growth and (bottom panel) relative levels of the nitrate-uptake proteins, NRT1 and NRT2 (per g dry root), in roots of tomato. Plants were grown at 28 °C/23 °C, 32 °C/27 °C, 36 °C/31 °C, or 40 °C/35 °C (day/night) for 15 days. Values are means ± 1 SE for four independent replicates. Different lowercase letters indicate significant difference among temperature treatments within each response variable.

As in the first main experiment (six days at 35 °C or 42 °C), root mass and root:shoot mass ratio decreased at high temperatures (15 days at 36 °C and 40 °C vs. 28 °C and 32 °C) (Figure 8). Also, as in the first experiment, the concentration of NRT1 and NRT2 decreased only at very high temperature (40 °C) (Figure 9).

## 3. Discussion

With increases in temperatures due to global warming, plants are likely to experience increasingly frequent, hotter, and longer episodes of abrupt heat stress (e.g., heat waves) in the future, and this will negatively impact plant function. Based on the limited past studies, we know that heat stress can negatively affect plant nutrient relations [11], but the effect of heat stress, chronic or abrupt, on root nutrient uptake rate has been little studied, excluding a few studies mostly using root pieces in vitro or heating only soil in intact plants, and we are aware of no previous research on effects of heat stress on nutrient-uptake proteins [7,19]. Our results show that in tomato, heat stress decreased total N content per plant [not shown, but = tissue N concentration (%N) × biomass (g), which both decreased, relative to control plants of the same age], by decreasing both plant growth (root growth more than shoot growth) and decreasing uptake rate of nutrients per g of root; similar negative effects of heat on total plant P, K, Fe, and B content were also observed [36].

Decreases in root growth and plant nutrient-uptake rate were likely caused by damage, rather than by lower available C for metabolism, given that heat stress increased root membrane damage [36] and %C, while decreasing root growth and protein concentration. The uptake rate of nutrients by roots decreased for most nutrients during severe, but not moderate heat stress, which was correlated with relative decreases in the concentration of nutrient-uptake proteins. It is also possible that decreases in the passive uptake and root-to-shoot transport of nutrients by transpiration-driven mass flow occurred during heat stress, and this contributed to decreases in plant nutrient uptake during heat stress. However, decreases in stomatal conductance were evident only at 42 °C on day 6, and though stomatal conductance was decreased then, transpiration was not lower due to increases in leaf-to-air vapor pressure differences. Further, results for B, which should be affected by mass flow more than the other mineral nutrients, given that it is smallest and uncharged, are not consistent with a heat-related decrease in passive nutrient uptake.

As in other previous studies [11], in roots and shoots subjected to the same high temperatures, roots were more sensitive to heat stress than shoots. In this study, both root and shoot growth decreased with heat stress, but the effect was larger for roots, as indicated by a decrease in root-to-shoot ratio (especially for severely-stressed plants). Also, photosynthesis (P_n_) was decreased by heat only on day 6 at 42 °C, while root membrane damage increased at both 35 °C and 42 °C [36]. Similarly, there was little effect of heat on shoot %C, but root %C increased with heat stress, and shoot %N decreased only at 42 °C, but decreased at both 35 °C and 42 °C in roots. The decrease in %N in shoots and roots with severe heat stress indicate that total plant N uptake was more impacted by heat than total plant growth. These results also indicate that heat likely did not impair C translocation from shoots to roots, otherwise, root %C would not increase, which is consistent with results of a study on abrupt heat stress in a heat-tolerant grass [35]. In addition, root protein content decreased with heat stress, which would result from an increase in protein degradation and/or decrease in protein synthesis, and either cause would constitute heat damage and both are known to occur during heat stress in roots [7].

Consistent with the effects of moderate vs. severe heat stress on total plant N content and %N, moderate heat stress did not affect the rate of nutrient uptake per g of root, but severe heat stress did decrease the uptake rate of four of five nutrients examined (N, P, K, Fe vs. B). Heat stress also decreased N-uptake rate per g root in the warm-season C_4_ grass, *Andropogon gerardii*, as measured by both sequential harvesting and ^15^N labeling [35]. Heat-related decreases in nutrient-uptake rates by roots can be caused by decreases in the concentration of nutrient-uptake proteins and/or by decreases in the activity (or transport or reaction rate) of individual uptake proteins. In this study, most of the eight nutrient-uptake proteins examined showed similar responses to heat stress; e.g., levels of the proteins per g root decreased compared to controls after 24 h of heat treatment at 35 °C and 42 °C (excluding NIP5;1), but after six days of heat treatment, only plants heated at 42 °C exhibited decreased levels of uptake proteins (excluding NIP5;1 and AMT1), and after seven days of post-heat recovery, levels of most uptake proteins had recovered to control levels (excluding FRO1, and non-significantly, KT1 and NRT1). Perhaps the heat tolerance of NIP5;1 vs. BOR1 and the other proteins is related to the fact that NIP5;1 is a channel protein, while BOR1 is not, and B is typically an uncharged molecule at physiological pH, but the other nutrients examined here are charged [37]. The effects of heat stress on the levels of the four N-assimilatory proteins examined were very similar to effects on nutrient-uptake proteins, though levels of all four proteins tended to remain slightly lower than controls after seven days of post-heat recovery. For the plasmalemma H^+^-ATPase, levels decreased during moderate and severe heat stress, but recovered within seven days. The dramatic difference we observed following one vs. six days of heat stress on the concentration of nutrient-uptake proteins (and nutrient-metabolism proteins, which showed the same pattern) indicates a need to examine the effects of heat stress at more time-points during heat treatments, to elucidate the detailed kinetics and mechanisms of plant responses to heat stress.

While this is the first study, to our knowledge, to examine effects of temperature on the levels of nutrient-uptake proteins in plants, other studies have examined effects of heat on the activity of nutrient-uptake or -assimilation proteins. For example, the uptake rate of P and K in detached corn root pieces during short-term incubations (thus reflecting heat effects on function, rather than concentration, of uptake proteins) increased up to 32 °C and 37 °C, with only small decreases at 37 °C and 42 °C, for K and P uptake, respectively [22]. Chopra [38] found that optimal activity of nitrate reductase in leaves of eight different crop species occurred at >45 °C during short-term incubations. In contrast, Hungria and Kaschuk [21] observed that chronic heat stress (18–38 days at 28 °C vs. 34 °C or 39 °C daytime) decreased the activities of nitrogenase, nitrate reductase, GS, and (especially) GOGAT, in leaves or nodules of the legume, *Phaseolus vulgaris*; heat also decreased the transport of N in xylem. Similarly, chronic heating (10–20 days at 20 °C vs. 35 °C daytime) decreased GS and GOGAT activities in fescue leaves (*Festuca arundinacea*) [39]. These limited results suggest that, unless temperatures reach extremely-high levels, heat stress likely decreases the activities of N metabolism proteins by decreasing their concentration in plant tissues, rather than inhibiting the function of individual N-metabolism proteins, consistent with results from our study.

## 4. Materials and Methods

### 4.1. Plant Growth Conditions, Temperature Treatments, and Harvesting

Seeds were planted in foam cubes and germinated in a controlled-environment chamber at 400–500 µmol·m^−2^·s^−1^ PAR (photosynthetically-active radiation), 14-h photoperiod, and 24 °C. Plants were watered daily and provided with starter nutrient solution (3 mM N, 1 mM Ca, 1 mM K, 0.5 mM P, 0.5 mM Mg, using Ca(NO_3_)_2_, K(NO_3_), KH_2_PO_4_, MgSO_4_; and 40 µM Fe, 6 µM Mn, 6 µM Zn, 40 µM B, 4 µM Cu, 0.1 µM Mo, using FeDTPA, MnCl_2_, ZnSO_4_, CuSO_4_, H_3_BO_3_, Na_2_MoO_4_).

After producing three to four post-embryonic leaves, plants were transferred to opaque aerated 4 L tubs with lids (one plant per tub) and grown hydroponically in complete nutrient solution [6.2 mM N, 2 mM Ca, 2 mM K, 2 mM Mg, 1 mM P, using NH_4_NO_3_, Ca(NO_3_)_2_, K(NO_3_), KH_2_PO_4_, MgSO_4_; and 71 µM Fe, 10 µM Mn, 10 µM Cl, 6 µM Zn, 6 µM Cu, 50 µM B, 0.1 µM Mo, using Fe DTPA, MnCl_2_, ZnSO_4_, CuSO_4_, H_3_BO_3_, Na_2_MoO_4_]. Solution pH was monitored daily and maintained at pH 5.6 with addition of 1 N KOH, and solution temperatures were monitored using a thermometer and were typically 3 °C lower than air temperature. Nutrient solution was changed every three to five days to maintain nutrient levels. Plants were moved daily to minimize positions effects inside the chamber. Chamber light, temperature, and CO_2_ levels were monitored continuously to ensure that chambers stayed at targeted conditions. We grew plants in hydroponics in order to minimize water stress during heat stress, and thus be able to ascribe treatment responses to heat alone, as well as to avoid confounding plant responses with rhizosphere influences, as would occur in soil.

In the main experiment, plants were grown under the above conditions for five days to allow for post-transfer acclimatization. Then plants (*n* = 4 per treatment combination, per harvest) were randomly assigned one of three temperature treatments: control = 20 °C/25 °C night/day, moderate heating = 30 °C/35 °C night/day, and severe heating = 37 °C/42 °C night/day (light and photoperiod as above). After six days of heat treatment, all plants were then moved to a single chamber and grown under control conditions for seven days to follow post-heating recovery. A random subset (*n* = 4) of plants was harvested after 24 h (1 day) and six days of heat stress, and after seven days of post-heating recovery. Day and night temperatures were both raised the same number of degrees during heat treatments, since climate-change projections indicate that global warming will affect both days and nights (and, in fact, will increase night, more than day, temperatures on average [4]). Our moderate heat treatment was intended to mimic a severe heat-wave under today’s climate, while the severe heat treatment was intended to mimic a severe heat-wave in the future, following significant global warming. In a second confirmatory experiment, plants were germinated and grown as above, but at the following temperatures and for 15 days before harvest: 28 °C/23 °C, 32/27, 36 °C/31 °C, and 40 °C/35 °C (day/night). At harvest, plants were separated into leaves, stems, and roots (roots after washing with DI water). Biomass was determined after oven drying at 70 °C for at least 48 h. Sub-samples of fresh root tissue for protein analysis were immediately frozen in liquid N_2_ after harvest and stored at −80 °C.

### 4.2. Leaf Gas-Exchange

To monitor the effects of heat treatment on both shoot metabolism and plant C assimilation, we measured steady-state net photosynthesis (P_n_; net CO_2_ exchange) immediately before harvesting, using an infrared gas analyzer (IRGA) (Model 6400, LiCOR, Lincoln, NE, USA) equipped with a 6-cm^2^ leaf-area cuvette which controlled environment conditions (CO_2_, light, and temperature); this system also simultaneously measures leaf transpiration, stomatal conductance to water vapor (G_s_), and internal CO_2_ concentration (C_i_). Gas exchange was measured on the most-recently-expanded attached leaf on day 1, and this same now-older leaf was measured on day 6; however, on day 13, the next-newest leaf was measured, since the previously-measured leaf was beginning to senesce, and this newer leaf was not yet fully-expanded. Measurements were made on leaves receiving direct light prior to measurement, at 370 ppm CO_2_, under saturating light (1500 µmol·m^−2^·s^−1^ PAR), and at the same temperature as plants were experiencing in the growth chamber (25 °C, 35 °C, or 42 °C during heating, 25 °C during recovery). Preliminary light-response curves were generated to determine optimal light levels during measurements and ensure that measurement light levels were not photoinhibitory.

### 4.3. Nutrient Relations

Total protein was extracted from tissue as in [40], by grinding 400 mg of frozen root tissue in liquid N_2_ in a mortar and pestle and then in 2 mL of extraction buffer of the following composition: 0.5 M Tris pH-8, 0.1 M potassium chloride, 0.9 M sucrose, 50 mM ethylene diamine tetra-acetic acid, 2% (*v*:*v*) β-mercaptoethanol, 10 µM leupeptin, and 1 mM phenyl methyl sulfonyl fluoride. The homogenate was transferred to a 15-mL tube, to which was added 2 mL volume of phenol, and then the tubes were incubated for 20 min at room temperature and centrifuged at 5600 rcf (relative centrifugal force) for 15 min at 4 °C to separate aqueous and organic phases. The upper phenol phase was recovered, and after addition of an equal volume of extraction buffer, centrifuged as above. Supernatant obtained was stored overnight at −20 °C in five volumes of 0.1 M ammonium acetate, to precipitate protein. Precipitated protein was then rinsed two times with ammonium acetate and three times with 80% acetone, followed by a final rinse with 100% acetone. Protein samples were dried and then re-solubilized in sample buffer (400 μL) containing 100 mM Tris pH 6.8, 0.5% (*v*/*v*) sodium dodecyl sulphate, and 1% (*v*/*v*) glycerol. Total protein concentration in the sample was determined using a colorimetric assay (DC Protein Assay, BioRad, Hercules, CA, USA), using bovine serum albumin as a standard.

The concentration of each macro- and micro- mineral nutrients was determined separately for powdered dry leaves, stems, and roots by the combustion-MS technique for C and N and by ICP-OES (Inductively Coupled Plasma Optical Emission Spectroscopy; model IRIS Intrepid II; Thermo Corp, Waltham, MA, USA) for remaining nutrients as in [41]. Total nutrient content in the entire plant was calculated from the concentration of each nutrient multiplied by the biomass of each tissue, and then the tissues were summed. Then the root specific uptake rate of each nutrient (x) (total g plant nutrient_x_ per g dry root per day) was calculated from the total amount of nutrient_x_ taken up during days 1–6 (heat stress) or from days 7–13 (recovery) (uptake during days 1–6 = total plant nutrient_x_ at day 6 minus total nutrient_x_ at day 1, uptake for days 7–13 = total nutrient_x_ at day 13 minus that at day 6).

The relative amount of nutrient-uptake proteins per unit total root protein was determined by quantitative ELISA (enzyme-linked immunosorbent assay), using protein-specific antibodies to oligopeptides of conserved domains of target proteins; conserved domains determined using bioinformatics techniques, as described in [40]. Antiserum specificity was confirmed with immune (western) blotting (immune vs. pre-immune serum, and antigen-purified vs. crude serum, in both tomato and arabidopsis), and pre-immune serum was used during ELISA to subtract signal attributable to non-specific binding. Then, the relative amount of each uptake protein per g root was calculated from total root protein per g root. Using commercially-available antibodies (Agrisera, Vännäs, Sweden) and ELISA, we also determined the relative level per unit root protein of the following key nutrient assimilatory proteins: NR (Nitrate Reductase), GOGAT (Glutamine OxoGlutarate Amino Transferase), GS (Glutamine Synthetase), and GDH1 (Glutamate DeHydrogenase), as well as the plasmalemma H^+^-ATPase, which generates the electrochemical gradient used for uptake of most nutrients.

### 4.4. Statistical Analysis

Experimental results were analyzed statistically using two-way (temperature x day) analysis-of-variance (ANOVA), with temperature and day as fixed factors (main experiment), or using one-way ANOVA with temperature as a fixed factor (second experiment) (Sigma-plot software, version 12). Following significant ANOVA results, the Holm-Sidak test was used to identify significant differences among the three temperature treatments. Treatment effects were considered significant if *p* < 0.05. Data were transformed (log-transformed for concentration and content data) if they did not meet assumptions of ANOVA (normality, equal variance), though this was rare, and results presented are untransformed means and error bars.

## 5. Conclusions

In summary, this study showed that abrupt or short-term heat stress can decrease root (relative to shoot) growth, the concentration of nutrient-uptake and -assimilation proteins, as well as total protein, in roots, and the rate of nutrient uptake by roots. Heat effects on roots and nutrient relations were often long lasting, with incomplete recovery in severely-stressed plants even after seven days of post-heat recovery. The relative effects of moderate vs. severe heat stress on plant nutrient content and concentration were correlated to different degrees with relative effects on root-to-shoot mass, nutrient-uptake rate per g of root, and levels of nutrient-uptake and N-assimilatory proteins. Notably, we did not examine the effects of heat stress on fruit yield or its nutritional quality, and future experiments should include these measures, since in tomato it is the fruit which is harvested for food.

Though heat stress was not accompanied by water stress in this study (as indicated by C_i_ results), in natural settings, heat stress is often accompanied by water stress caused by drought, high leaf transpiration, or decreases in xylem hydraulic conductivity, and water stress may also contribute to decreases in nutrient uptake during heat stress (e.g., by decreasing water uptake or transport from roots to shoots) [12]. So, increases in abrupt or short-term heat stress with global warming in the future will likely have overall negative effects on plant nutrient relations that will become more severe as temperatures rise, which will contribute to decreases in both crop productivity, as well as nutritional quality. Efforts to develop crop genotypes which maintain nutrient uptake and assimilation during heat stress will likely need to include a focus on increasing the thermotolerance of both root growth and protein synthesis, including synthesis of nutrient-metabolism proteins.
